# Factors associated with the quality of life of subjects with facial disfigurement due to surgical treatment of head and neck cancer

**DOI:** 10.4317/medoral.22072

**Published:** 2018-02-25

**Authors:** Túlio E. Nogueira, Marcelo Adorno, Elismauro Mendonça, Cláudio Leles

**Affiliations:** 1 DDS, MSc; Department of Prevention and Oral Rehabilitation, School of Dentistry, Federal University of Goias, Goiania, Goias, Brazil; 2 DDS, Department of Prevention and Oral Rehabilitation, School of Dentistry, Federal University of Goias, Goiania, Goias, Brazil; 3 DDS, MSc, PhD; Department of Oral Medicine, School of Dentistry, Federal University of Goias, Goiania, Goias, Brazil; 4 DDS, MSc, PhD, Department of Prevention and Oral Rehabilitation, School of Dentistry, Federal University of Goias, Goiania, Goias, Brazil

## Abstract

**Background:**

Facial disfigurement has been considered one of the most challenging consequences of the surgical treatment for head and neck cancer patients, mainly due to the importance of the facial region for the personal identity, body self-image and interpersonal interactions, which might affect negatively the quality of life. The aim of this study was to assess factors associated with the quality of life of subjects with facial disfigurement due to surgical treatment.

**Material and Methods:**

Clinical data were retrieved from 103 patient’s medical records and quality of life data were collected using the Functional Assessment of Cancer Therapy (FACT-H&N) questionnaire. Moreover, the degree of facial disfigurement was classified by means of a specific ordinal scale.

**Results:**

Data from the FACT-H&N questionnaire showed that the domain directly related to head and neck symptoms was considered the most impacted, while emotional domain was the least affected. Lower quality of life was associated with sequels in the neck and/or lower third of the face (β=-0.39; *p*=0.001), a higher level of disfigurement (β=-0.29; *p*=0.016) and female gender (β=-0.20; *p*=0.038).

**Conclusions:**

Disfigurement due to surgical treatment was significantly associated with the functional dimension of the patients, especially in extensive sequels in the cervical and lower regions of the face.

** Key words:**Quality of life, Head and neck cancer, Patient-reported outcomes.

## Introduction

Head and neck cancer (HNC) involves malignant neoplasms located in the mucosal surfaces of the upper aerodigestive tract, including the oral cavity, pharynx, larynx, and paranasal sinuses, as well as cancers of the major and minor salivary glands (1). Squamous cell carcinoma (SCC) is the most common histological type comprising about 90% of the cases (2). Depending on the TNM stage of the tumour, treatment for HNC may vary from isolated surgical approaches in initial stages to combined surgery, chemotherapy and radiotherapy in advanced cases (3).

Extensive resections of the tumour and of the related structures are frequently required in surgical cases, affecting patient’s orofacial functioning, appearance and consequently quality of life (QoL) (4). These problems are also aggravated by the intrinsic morbidity and mortality of the disease, which are commonly associated with feelings such as high suffering, depression, anxiety, hopelessness, fear, uncertainty about the future, dissatisfaction and other negative reactions (5,6).

In most patients, sequels of the surgical treatment are both functional and aesthetic and facial disfigurement has been considered one of the most challenging consequences, mainly due to the importance of the facial region for the patient’s personal identity, body self-image and interpersonal interactions (5,7). In addition, such disfigurement is often highly visible and not easily hidden from view (8).

The use of specific questionnaires and measures to assess such impact are essential tools for the comprehensive understanding of this population and the information obtained from QOL studies has the potential to be incorporated in the clinical practice to improve the quality of care (9).

This study aimed to assess factors associated with the quality of life of subjects with facial disfigurement due to surgical treatment of head and neck cancer. It was hypothesised that the extent and location of the surgical sequels might be associated with the levels of quality of life impacts.

## Material and Methods

This cross-sectional study was carried out in a regional reference hospital for cancer treatment (Araujo Jorge Hospital, Goiania, Goias, Brazil). Data collection occurred between May 2013 and April 2014. The research protocol was previously approved by the local Research Ethic Committee (protocol 169/11) and a written informed consent was obtained from all individual participants included in the study.

The target population comprised patients undergoing treatment for HNC who met the following inclusion criteria: 18 years-old or older; presence of visible disfigurement in the head and/or neck region due to resective surgery as part of cancer therapy, identified by the study investigators through direct visual inspection. Surgical resection must have occurred at least 6 months previously and subjects should be able to communicate efficiently during data collection. There were no restrictions regarding histopathologic diagnosis, clinical status and treatment stage. However, individuals in terminal illness or presenting major general health impairments, as well as patients treated with maxillofacial prosthesis were excluded from the study sample.

Potentially eligible participants were recruited through convenience sampling. They were invited to participate through direct approach in the hospital setting during follow-up visits. Before agreement to participate, all subjects received a brief description of the study, including its objectives, methods and time commitment.

Two previously calibrated investigators conducted data collection. Clinical data were retrieved from patient’s medical records and comprised information about clinicopathological diagnosis, previous treatment procedures and current disease status. Quality of life was assessed by means of a face-to-face interview using the Functional Assessment of Cancer Therapy (FACT-H&N) questionnaire, a specific instrument to assess quality of life impacts of HNC patients (10).

The FACT-H&N questionnaire is a multidimensional instrument that consists of 27 core items which assess four domains (physical, social and family, emotional, and functional well-being), complemented by 12 head and neck-specific items (named “additional concerns”). Responses are rated on a 0-4 Likert scale, and scores are combined to give subscale scores for each domain and a global score. Higher scores represent better quality of life (10).

In addition, we classified the degree of facial disfigurement of each patient based on the scale proposed by Katz *et al.*, which is a single-item, ordinal scale ranging from “1” (minimal disfigurement) to “9” (maximum disfigurement). Ratings take into account the size of the disfigured area, the degree of face/neck shape distortion, the extent of impairment in facial expression, and the visibility of the disfigured area (8).

Descriptive statistics was performed using frequency analysis for nominal variables, and central tendency and dispersion measures for continuous variables. T-test and ANOVA were used for group comparisons, and stepwise multiple linear regression was used to test the association between QoL data and the independent variables. Statistical significance was set at *p*<0.05 and MedCalc Statistical Software version 13.1.2 (MedCalc Software BVBA, Ostend, Belgium) was used for data analysis.

## Results

**Figure 1:**
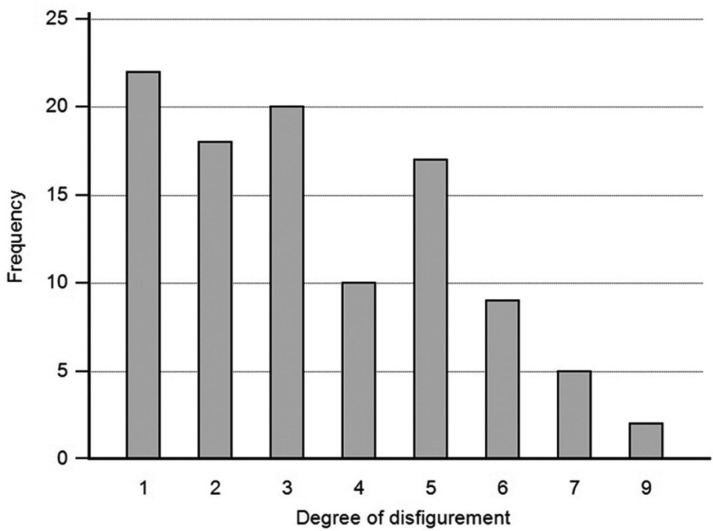
Frequency distribution of the degrees of disfigurement according to the classification by Katz *et al.* (9) (2000).

The final sample comprised 103 individuals, with 78.6% being male, and ages ranging from 20.0 to 81.6 years-old (mean age=56.7; SD=13.1). The main characteristics of the sample are described in  Table 1, and the distribution regarding the classification of facial disfigurement is shown in Figure 1.

**Table 1:**
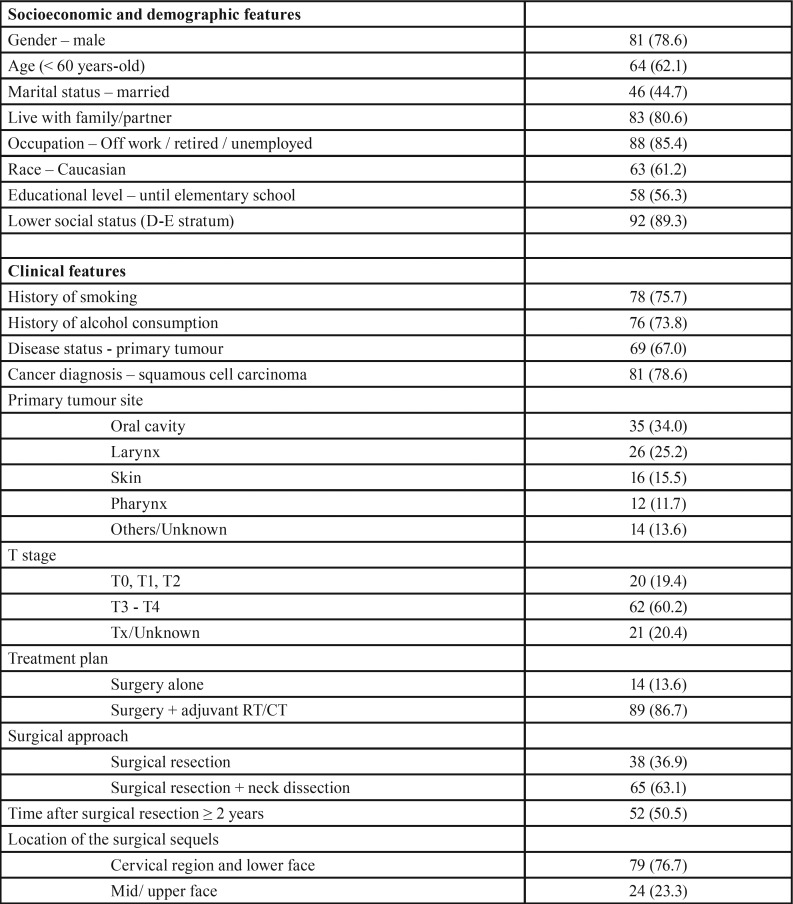
Frequency of the main characteristics of the study sample (percentage in parentheses).

 Table 2 summarises data from the FACT-H&N questionnaire, considering global and domain scores. “Additional concerns” presented a mean score that was only 56% of the highest possible score. This domain, which is directly related to “head and neck” symptoms, was then considered the most impacted. In contrast, emotional domain was the least affected (79.1% of the maximum possible score).

**Table 2:**
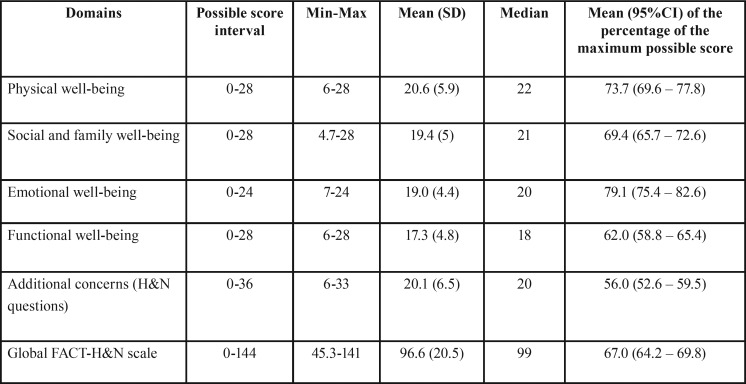
Results of FACT-H&N scores (domains and global scale).

**Figure 2:**
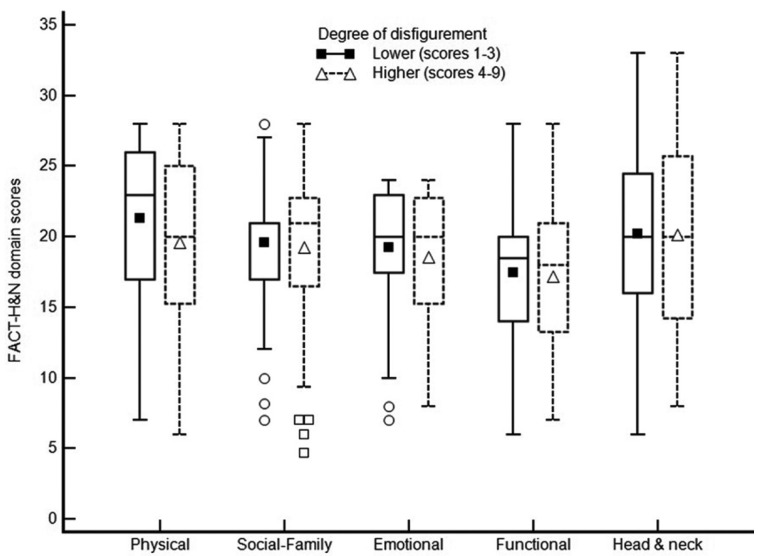
FACT-H&N scores according to the disfigurement level and the scale domains. Central value markers represent mean values.

FACT-H&N scores’ distribution according to the degree of disfigurement and the scale domains is presented in Figure 2. For this analysis, the degree of disfigurement was dichotomised into lower (1 to 3) and higher degree (4 to 9), in order to improve interpretability of results. In the bivariate analysis, values of FACT-H&N domain scores were not influenced by the degree of disfigurement (*p*>0.05). On the other hand, overall FACT-H&N mean scores were lower for females (*p*=0.025), subjects with lower education level (*p*=0.013), those who reported a smoking history (*p*=0.002) and who received adjuvant chemotherapy and radiotherapy (*p*=0.030). QoL was also influenced by the location of the surgical sequel in the lower third and cervical region (*p*<0.05). No other clinical and sociodemographic variables showed significant association with QoL measures.

Each potentially relevant sociodemographic and clinical variable identified on bivariate analysis was tested for its association with FACT-H&N scores using single linear regression, followed by multiple regression analysis. The final regression model ( Table 3) between quality of life measures (overall FACT-H&N scale and domains) and independent clinical and sociodemographic factors revealed that the reduction in the quality of life was associated with the location of the sequel (worse scores in the neck/lower third of the face (β=-0.39; *p*=0.001), higher level of disfigurement (β=-0.29; *p*=0.016) and female gender (β=-0.20; *p*=0.038).

**Table 3:**
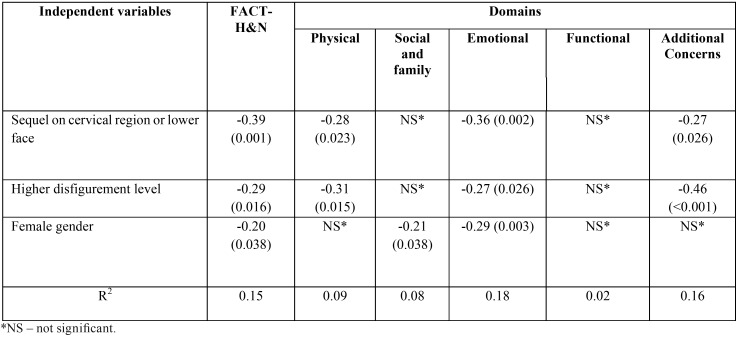
Results of the multiple linear logistic regression for scores of FACT-H&N and domains. Data are expressed as standardised regression coefficients (and *p*-value in parenthesis).

## Discussion

This study aimed to assess the effect of facial disfigurement on quality of life of patients that underwent surgery as part of the HNC therapy, as well as the influence of clinical and sociodemographic variables on patient outcomes.

Results showed that facial disfigurement was significantly associated with the physical, emotional and additional concerns’ domains of the FACT-H&N questionnaire, especially in cases of extensive sequels in the cervical and lower regions of the face. It was also observed that the association between facial disfigurement and quality of life was significantly greater in women, concerning the social and familiar, functional and head and neck cancer specific domains. Similar findings were reported in the study of Wells *et al.* which assessed predictors of QoL in HNC survivors and concluded that younger age, lower socio-economic status, unemployment and self-reported comorbidity contributed to poorer generic and cancer-specific QoL (11).

The impact of the sequels of HNC treatment in patient’s quality of life has been widely assessed by a variety of specific instruments. A recent systematic review analysed 57 QoL instruments and no gold standard was set because of the large volume and heterogeneity of measures that can be the target for quality of life assessment (12). We employed the FACT-H&N questionnaire, a multidimensional instrument that allows a broad measurement of how the patient´s condition affects physical health, psychological state, level of independence, social relationships and the relations between individuals and environmental characteristics (13). Although this questionnaire was originally conceived as a self-administered instrument, we adopted the interview approach since the sample presented limiting characteristics related to sociocultural aspects and cognitive level.

Some limitations can be addressed in our study such as the cross-sectional design that, despite being frequently adopted in similar studies, reduces the extent of interpretation of findings and precludes quality of life analysis in relation to different stages of the disease course. In addition, there is limited evidence of inter-rater reliability of the measures of the degree of disfigurement obtained by the rating scale used in this study.

The majority of the sample was comprised of male subjects (78.6%), with a mean age of 56.7 years (20-81.6) and presented low socioeconomic status (89.3% classified in the lowest stratum according to the Brazilian Economic Classification Criteria), which is similar to sample characteristics found in other Brazilian studies (14-16). The association between low socioeconomic status and the occurrence of HNC was also evidenced in a systematic review by Boing and Antunes (17).

In addition, 75.7% of the participants reported smoking history and 73.8% declared alcohol consumption, both of which are important aetiological factors associated with HNC. Moreover, the sample presented a low educational level, as 56.3% of the subjects were illiterate or did not complete elementary school. In our investigation, higher FACT-H&N scores were achieved by individuals with greater educational level (*p*=0.013). Higher educational levels generally ensure better jobs, with labour support as medical license and retirement, which could explain such differences among scores.

Despite the wide range of studies on this topic, which have focused on different approaches concerning sample characteristics, study designs and selected outcomes, the implications of these findings are still poorly discussed and managed on clinical routine (12). In this sense, quality of life assessment in HNC patients should be incorporated into clinical practice in order to contribute to an individualised therapeutic plan and to improve the quality of care offered to patients and their families. Moreover, the importance of a multidisciplinary approach and comprehensive care in HNC patients is noteworthy, as highlighted in previous studies (18,19).

There is no strong evidence of a linear relationship between the level of facial disfigurement and dysfunction and impacts on QoL (20), which suggests that other emotional and psychosocial factors may play a relevant role on patient’s individual response. Consequently, a set of resources is essential for the development of an effective multidisciplinary approach for the HNC patient, including material, financial and human resources. In Brazil, there is a marked scarcity in the provision of proper management of facial surgical sequels in most of the oncology centres, especially in the public health system. There is a limited offer of oral and maxillofacial rehabilitation facilities, mainly due to the lack of specialists and skilled professionals in this area. Currently, there are around 70 registered specialists in the whole country according to the Federal Council of Dentistry, and most of these professionals are not fully incorporated into a multidisciplinary healthcare team for the management of HNC patients.

## Conclusions

Head and neck disfigurement due to cancer surgical treatment was significantly associated with the functional dimension of patient’s QoL, especially in extensive sequels in the cervical and lower regions of the face. The emotional dimension affected women more strongly than men.

## References

[1] Lydiatt WM, Patel SG, O'Sullivan B, Brandwein MS, Ridge JA, Migliacci JC (2017). Head and Neck cancers-major changes in the American Joint Committee on cancer eighth edition cancer staging manual. CA Cancer J Clin.

[2] Döbrossy L (2005). Epidemiology of head and neck cancer: Magnitude of the problem. Cancer Metastasis Rev.

[3] Martino R, Ringash J (2008). Evaluation of quality of life and organ function in head and neck squamous cell carcinoma. Hematol Oncol Clin North Am.

[4] Murphy BA, Ridner S, Wells N, Dietrich M (2007). Quality of life research in head and neck cancer: A review of the current state of the science. Crit Rev Oncol Hematol.

[5] Costa EF, Nogueira TE, de Souza Lima NC, Mendonça EF, Leles CR (2014). A qualitative study of the dimensions of patients' perceptions of facial disfigurement after head and neck cancer surgery. Spec Care Dentist.

[6] Fingeret MC, Hutcheson KA, Jensen K, Yuan Y, Urbauer D, Lewin JS (2013). Associations among speech, eating, and body image concerns for surgical patients with head and neck cancer. Head Neck.

[7] O'Brien K, Roe B, Low C, Deyn L, Rogers SN (2012). An exploration of the perceived changes in intimacy of patients' relationships following head and neck cancer. J Clin Nurs.

[8] Katz MR, Irish JC, Devins GM, Rodin GM, Gullane PJ (2000). Reliability and validity of an observer-rated disfigurement scale for head and neck cancer patients. Head Neck.

[9] Vartanian JG, Rogers SN, Kowalski LP How to evaluate and assess quality of life issues in head and neck cancer patients. Curr Opin Oncol.

[10] List MA, D'Antonio LL, Cella DF, Siston A, Mumby P, Haraf D (1996). The performance status scale for head and neck cancer patients and the functional assessment of cancer therapy-head and neck scale. A study of utility and validity. Cancer.

[11] Wells M, Swartzman S, Lang H, Cunningham M, Taylor L, Thomson J (2016). Predictors of quality of life in head and neck cancer survivors up to 5 years after end of treatment: a cross-sectional survey. Support Care Cancer.

[12] Ojo B, Genden EM, Teng MS, Milbury K, Misiukiewicz KJ, Badr H (2012). A systematic review of head and neck cancer quality of life assessment instruments. Oral Oncol.

[13] he Whoqol Group (1995). The World Health Organization quality of life assessment (WHOQOL): position paper from the World Health Organization. Soc Sci Med.

[14] de-Jesus RR, Meyer TN, Leite IC, Pereira AA, Armond MC (2010). Epidemiologic profile and quality of life of patients treated for oral cancer in Juiz de Fora, Minas Gerais, Brazil. Med Oral Patol Oral Cir Bucal.

[15] Machado BCP, Gonçalves LM, Júnior JRSB, Cruz MCFN (2009). [Quality of life evaluation of patients with head and neck cancer in Maranhão State]. Rev Bras Pesq Saúde.

[16] Melo Filho MR, Rocha BA, Pires MBO, Fonseca ES, Freitas EM, Junior HM (2013). Quality of life of patients with head and neck cancer. Braz J Otorhinolaryngol.

[17] Boing AF, Antunes JLF (2011). [Socioeconomic conditions and head and neck cancer: a systematic literature review]. Cien Saude Colet.

[18] Fingeret MC, Teo I, Epner DE (2014). Managing body image difficulties of adult cancer patients: lessons from available research. Cancer.

[19] Fingeret MC, Yuan Y, Urbauer D, Weston J, Nipomnick S, Weber R (2012). The nature and extent of body image concerns among surgically treated patients with head and neck cancer. Psychooncology.

[20] Vickery LE, Latchford G, Hewison J, Bellew M, Feber T (2003). The impact of head and neck cancer and facial disfigurement on the quality of life of patients and their partners. Head Neck.

